# Efficacy of vitamin D supplementation on COPD and asthma control: A systematic review and meta-analysis

**DOI:** 10.7189/jogh.12.04100

**Published:** 2022-12-16

**Authors:** Yuhang Wang, Jin Wang, Li Chen, Huan Zhang, Ling Yu, Yulong Chi, Mengli Chen, Yun Cai

**Affiliations:** 1 Center of Medicine Clinical Research, Department of Pharmacy, Medical Supplies Center of PLA General Hospital, Beijing, China; 2 Department of Information, PLA General Hospital, Beijing, China; 3 Laboratory of Department of Pulmonary and Critical Care Medicine, PLA General Hospital, Beijing, China; 4 Department of Pharmacy, Medical Supplies Center of PLA General Hospital, Beijing, China

## Abstract

**Background:**

The role of vitamin D (VD) in the management of chronic obstructive pulmonary disease (COPD) and asthma remains largely undetermined. In the present meta-analysis, we aimed to comprehensively investigate the efficacy of VD in the treatment of COPD and asthma according to the latest update.

**Methods:**

The PubMed, Embase, and Cochrane Library databases were searched from their inception to June 2, 2022. Randomized controlled trials (RCTs) comparing the efficacy of VD with placebo against COPD or asthma were included.

**Results:**

A total of 11 RCTs consisting of 1183 COPD patients and 19 RCTs consisting of 2025 asthmatic patients were finally included. As for pulmonary function, FEV1/FVC was not changed significantly, while FEV1% was improved in the VD group. In the asthma subgroup, FEV1% was not changed significantly, while FEV1/FVC was improved in the VD group. For the questionnaire and rating scale, the mMRC (modified Medical Research Council) dyspnoea scale score for COPD and ACT (Asthma Control Test) score for asthma were not significantly changed, while the SGRQ (St. George′s Respiratory Questionnaire) score for COPD was improved in the VD group. For inflammation indicators, IL-6 and IL-10 were statistically equivalent between the VD and placebo groups, while IgE, IL-5, and IL-10 (baseline VD deficiency subgroup) were improved in the VD group. The exacerbation, length of hospital stays, and mortality were statistically equivalent between the two groups.

**Conclusions:**

VD supplementation improved the indicators of asthma and COPD, especially in pulmonary function, SGRQ scores, IL-5, and IgE.

**Registration:**

The protocol could be found at PROSPERO with the registration number of CRD42020218058.

Chronic obstructive pulmonary disease (COPD) has the highest mortality rate among chronic respiratory diseases [1]. COPD patients often show incomplete reversibility of airflow obstruction caused by emphysema and chronic bronchitis. It can eventually develop into severe diseases, such as pulmonary heart disease and respiratory failure. Currently, there is no good way to prevent development of the disease. Similarly, asthma is another common chronic inflammatory disease that can start at a young age. Like COPD, asthma can also develop into chronic airway limitation because of uncontrolled inflammation [2]. Because inflammation is crucial in pathogenesis of asthma, inflammation control is primary goal of asthma control [3]. Drug therapy includes bronchodilators, glucocorticoids and sometimes antibiotics. However, long-term use of above-mentioned drugs triggers plenty of adverse events. In addition to commonly used drugs, Vitamin D (VD) is currently considered to be very promising for its efficacy and excellent tolerance.

VD receptor (VDR) is a transcription factor that affects expressions of thousands of genes. Besides its function in mineral metabolism and skeletal health, it may play an important role in other functions, such as the physiology of immune system, glucose metabolism and neurocognitive functions. [4,5] Airway epithelial cells and immune cells in lung express VDR, and regulatory mechanism of the activity of 25(OH)D 1α-hydroxylase enzyme in lung is different from that in the kidney, which may lead to the increase of 1,25(OH)2D in the lung, resulting in changes of immune regulation [6]. Some studies support the correlation between serum 25(OH)D and the severity of COPD. In a meta-analysis consisting of 27 128 participants, the serum 25(OH)D level is positively correlated with pulmonary function parameters, such as forced expiratory volume in one second (FEV1) and forced vital capacity (FVC) [7]. As for COPD risk, severity, and exacerbation, a meta-analysis shows a negative correlation with serum 25(OH)D levels [8]. Similar results have been observed in asthmatic patients [9]. Therefore, serum 25(OH)D levels may affect asthma and COPD control. However, it remains largely unknown whether VD supplementation can improve the disease state.

To date, the results of current meta-analysis of VD supplementation in controlling COPD and asthma are inconsistent. Two new randomized controlled trials (RCTs) for COPD and seven RCTs for asthma are available. Considering that VD supplementation may be a low-cost, low-risk method of controlling asthma and COPD, we conducted the present meta-analysis and aimed to comprehensively investigate efficacy of VD in the treatment of COPD and asthma control according to the latest update.

## METHODS

### Literature search

The PubMed, Embase, and Cochrane Library databases from their inception up to June 1, 2022 were independently searched by two investigators (Y.H.W and J.W.). The term used for search strategy was (“COPD[Title/Abstract]” OR “asthma [Title/Abstract]” AND “vitamin D[Title/Abstract]”).

### Study selection

Trials were included if their participants were patients with COPD or asthma. Trials were considered to be eligible if they compared VD supplementation at any dose with the placebo. Studies were included if they reported one or more of the outcomes. Efficacy-related outcomes included length of hospital stay, mortality, FEV1, FEV1/FVC, exacerbations, SGRQ (St. George′s Respiratory Questionnaire) scores, mMRC (modified Medical Research Council) dyspnoea scale scores, ACT (Asthma Control Test) scores, cytokines, IgE, and eosinophil counts. Studies were excluded if they were reviews, conference abstracts, editorials or case reports. Studies conducted on healthy people, animals or in vitro models were also excluded.

### Data extraction and evaluation

To determine the eligibility of identified trials, the titles and abstracts were independently screened by two authors (Y.H.W and J.W). Full texts were obtained when necessary. Any disagreements were resolved by a third investigator (Y.C). A final consensus was reached among all investigators. The relevant data were independently extracted by two investigators (Y.H.W and J.W), and the risk of bias was also assessed. The Cochrane assessment tool was used to evaluate the quality of each included study.

VD deficiency was defined as serum 25(OH)D≤20 ng/mL [10]. mMRC ranged from 0 to 4, with higher scores indicating more severe dyspnoea. SGRQ scores decreasing at least four points in the total score were defined as a clinically significant improvement in quality of life. An increase in ACT/CACT (Childhood Asthma Control Test) value indicated better asthma control. The GOLD (Global Initiative for Chronic Obstructive Pulmonary Disease) stage is an intuitive system for classifying COPD severity based on FEV1, ranging from I (FEV1 ≥ 80%) to IV (FEV1 < 30%) [11].

### Statistical analysis

All analyses were carried out using the Review Manager program, version 5.3. The heterogeneity of study results was assessed by the χ^2^ test, and the inconsistency was determined by the *I^2^* measure. Subgroup analysis was used to explore possible causes of heterogeneity among study results. Mean differences (MD) and standardized mean differences (SMD) were used for continuous variables, while odds ratios (ORs) were used for dichotomous variables. Der Simonian-Laird random-effects (χ^2^ test *P* ≤ 0.10) or Mantel-Haenszel fixed-effects (χ^2^ test *P* > 0.10) model was used for ORs, and 95% confidence intervals (CIs) were used throughout the meta-analysis. The significance of the pooled ratios was determined by Z-test, and a *P* value of <0.05 was considered statistically significant. In present study, the mean and standard deviation were calculated by estimating the extreme value and quartile spacing according to the Cochran handbook and Wan’s method [12].

## RESULTS

### Included studies

**Figure 1:**
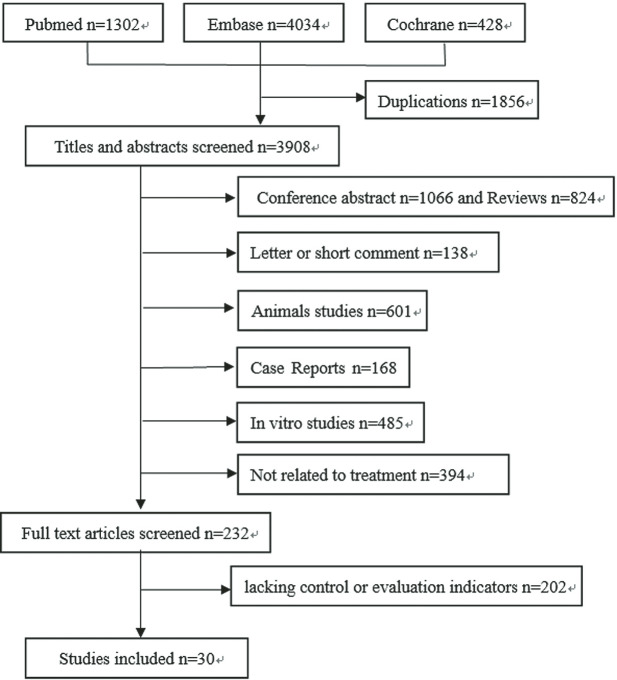
Flowchart of the article selection process.

A total of 3908 citations were identified from the three databases by literature search after the duplications were removed. Reviews, case reports, conference abstracts, and editorials, in vitro or animal studies were excluded by reading the abstract, and 232 potentially relevant full-text articles were screened. Moreover, 139 articles were excluded due to the lack of control or evaluation indicators [13-42]. **Figure 1** illustrates the detailed search and study selection process.

### Study characteristics

Eleven RCTs consisting of 1183 COPD patients and 19 RCTs consisting of 2025 asthmatic patients were finally included in the present meta-analysis. Nine RCTs of asthma were for children. **Table 1** and **Table 2** list main characteristics of the studies included in analysis. The quality of RCTs was evaluated by the Cochrane risk of bias tool, and results showed that the quality of these RCTs was high (Figure S1 in the **Online Supplementary Document**).

**Table 1:** Basic characteristics of included studies for chronic obstructive pulmonary disease (COPD)

Author, Year	Region	Design	Group (VD/placebo)											VD dose	Evaluation time	Outcomes*
			**Sample size**	**Age (years)**	**BMI**	**CS**	**GOLD stages**				**FEV1/FVC baseline**	**Baseline VD, ng/ml (m ± SD)**	**VD, ng/ml (m±SD) at EOT**			
Rafiq, 2022 [42]	Netherlands	RCT	74/81	65 ± 9/67 ± 9	28.1 ± 5.1/27.4 ± 5.4	25/23	I	5.3/6.2			45 ± 12/43 ± 14	38 ± 15/40 ± 17		16 800 IU once a week for 1 y	12 mo	1
							II	44/42								
							III	41.3/37								
							IV	9.3/14.8								
Dastan, 2019 [13]	Iran	RCT	33/34	64.42 ± 7.58/63.24 ± 8.41	21.03 ± 1.97/20.27 ± 1.67	6/9	II	15/16				10.59 ± 3.90/11.25 ± 3.09	18.17 ± 4.24/11.35 ± 3.16	300 000 IU single injection	6 d, 30 d	5,8,9,10
							III	12/13								
							IV	6/5								
Alavi Foumani, 2019 [14]	Iran	RCT	32/31	67.9 ± 7.9/68.4 ± 7.8	24.33 ± 2.13/24.55 ± 1.94						57.43 ± 12.09/58.9 ± 9.56	19.33 ± 5.18/18.55 ± 4.58	51.83 ± 7.93/19.43 ± 5.22	50 000 IU once a week for 8 weeks, then once a month for 4 mo	2 mo, 6 mo	1,3,4
Pourrashid, 2018 [15]	Iran	RCT	30/32	62.7 ± 8.26/64.0 ± 8.77	22.99 ± 1.69/22.90 ± 1.97	7/7	II		13/16			10.82 ± 3.73/11.01 ± 2.99	36.85 ± 11.80/12.30 ± 3.66	300 000 IU single injection	30 d, 4 mo	5,6,8,9
							III		11/12							
							IV		5/5							
Rafiq, 2017 [16]	Netherlands	RCT	24/26	64/61	29.6 ± 6.7/26.4 ± 5.1	18/18	I		6/4		48.76 ± 15.01/48.46 ± 12.51	16.95 ± 6.09/16.27 ± 6.81	38.45/21.2	1200 IU daily for 6 mo	3 mo, 6 mo	3,4
							II		8/14							
							III		8/5							
							IV		2/3							
Khan, 2017 [17]	Pakistan	RCT	60/60	46.28 ± 8.83	22.57 ± 1.72							24.08 ± 2.58	29.6 ± 8.74	2000 IU daily for 6 mo	2 mo, 4 mo, 6 mo	1
Sanjari, 2016 [19]	Iran	RCT	39/42	55.8 ± 9.5/58.4 ± 9.5	-	-						23.6 ± 10.82/24 ± 10.42	39.14 ± 20.91/26.12 ± 15.71	50 000 IU VD daily for seven days	8 d	3,4
	Iran	RCT	39/42	55.6 ± 10.4/58.4 ± 9.5	-	-						22 ± 13.98/24 ± 10.42	22.88 ± 17.79/26.12 ± 15.71	100 IU calcitriol daily for 7 d		
Zendedel, 2015 [18]	Iran	RCT	44/44	<45 (4.5%)/43 (97.7%)	-	-						-	-	100 000 IU per month, for 6 mo	6 mo	3
Martineau, 2015 [20]	UK	RCT	122/118	64.8 ± 7.9/64.5 ± 9.2	27.9 ± 6.1/27.2 ± 6.7	56/42	I		32/39			18.19 ± 11.18/18.71 ± 9.33	27 ± 11.02/18.87 ± 10.78	2-moly 120 000 IU for a year	12 mo	1,3,6,9,10
							II		57/56							
							III		25/27							
							IV		8/6							
Bjerk, 2013 [21]	USA	RCT	18/18	67.6 ± 7/68 ± 8	-	7/11					61 ± 13/56 ± 17	22.6 ± 10.5/24.4 ± 10.5	32.6 ± 8.2/22.1 ± 10.1	2000 IU daily for 6 weeks	6 weeks	6
Lehouck, 2012 [22]	Belgium	RCT	91/91	68 ± 9/68 ± 8	25 ± 5/24 ± 5	13/19	II		25/24			20 ± 12/20 ± 11	52 ± 16/22 ± 13	100 000 IU every 4 weeks for 1 y.	12 mo	1,9
							III		43/48							
							IV		23/19							

CS – current smoker, BMI – body mass index, VD – vitamin D, RCT – randomized controlled trial, EOT – end of treatment, COPD – chronic obstructive pulmonary disease, m ± SD – mean ± standard deviation, d – day, mo – month, y – year

*1 – exacerbations for COPD, 2 – exacerbations for asthma, 3 – FEV 1, 4 – FEV1/FVC, 5 – mMRC scores, 6 – SGRQ scores, 7 – ACT/CACT scores, 8 – length of hospital stay, 9 – mortality, 10 – cytokines, 11 – IgE, 12 – eosinophils.

**Table 2:** Basic characteristics of included studies for asthma

Author, year	Region	Design	Group (VD/placebo)								VD dose	Evaluation time	Outcomes*
			**Sample size**	**Ages**	**BMI**	**SE**	**ACT/ CACT score**	**FEV1/FVC baseline**	**Baseline VD, ng/ml (m ± SD)**	**VD, ng/ml (m ± SD) at EOT**			
Thakur, 2021 [23]	India	RCT	28/28	9 ± 1.7/8.7 ± 1.6	-0.90/-0.83 (z score)		18 ± 2.9/15.5 ± 2.7		15.8 ± 8.2/16.5 ± 9.9	35.47 ± 10.0/18.78 ± 6.6	2000 IU daily for 10 d	3 mo	2,3
Jat, 2020 [24]	India	RCT	125/125	8.2 ± 2.3/7.8 ± 2.2		24/21	21.7 ± 4.2/21.9 ± 3.6	98.5 ± 10.9/99.3 ± 10.1	11.6 ± 4.6/10.8 ± 4.4	18.1 ± 7.1/12.0 ± 6.0	1000 IU daily for 9 mo	9 mo	2,3,4,7
Forno, 2020 [25]	USA	RCT	96/96	9.9 ± 2.5/9.7 ± 2.5	0.9 ± 1.1/0.9 ± 1.3 (z score)	25/22	22.0 ± 3.2/21.3 ± 3.6	91.5 ± 9.3/89.6 ± 10.1	22.5 ± 4.6/22.8 ± 4.6	49.4/24.6	4000 IU daily for 48 weeks	48 weeks	2
Andujar-E, 2020 [26]	Spain	RCT	53/53	54.57 ± 15.83/56.61 ± 15.00	28.21 ± 5.23/29.83 ± 7.41	3/4	17.71 ± 4.54/19.02 ± 4.59	76.99 ± 7.84/78.40 ± 7.73	16.71 ± 6.71/17.48 ± 5.72	58.72 ± 28.69/17.38 ± 6.83	16 000 IU per week for 6 mo	6 mo	2,3,4,11
Shabana, 2019 [27]	Egypt	RCT	42/37	34.00 ± 7.40/35.50 ± 7.00	25.15 ± 5.75/26.68 ± 2.82	0/0		63.21 ± 10.95/64.41 ± 7.90	17.56 ± 2.74/18.16 ± 2.89	25.00 ± 2.87/17.97 ± 3.21	single dose of 300 000 IU	3 mo	3,4,10
Dodamani, 2018 [28]	India	RCT	15/15	33 ± 12.5/32 ± 12.2				69.7 ± 10.7/66.3 ± 13.8	22.68 ± 10.27/19.83 ± 10.49	38.7 ± 12.5/34.6 ± 24	60 000 IU once weekly for 8 weeks	2 mo, 4 mo, 6 mo	2,10
Ramos-M, 2018 [29]	Mexico	RCT	43/43	41 ± 11/42 ± 15							100 IU daily for 6 mo	6 mo	10,11,12
Ali, 2017 [30]	Egypt	RCT	32/28	43/48 (median)	30.07/34.1 (median)			82/85 (median)	21.18 ± 10.33/23.8 ± 12.8	22.6/16.3 (median)	400 IU daily for 4 mo	4 mo	3,4
Tachimoto, 2016 [31]	Japan	RCT	54/35	10.0 ± 2.4/9.8 ± 2.2	17.6 ± 2.6/17.4 ± 2.9		23 (23-25)/24.5 (24-25) 25 (23-27)/26 (25-27)	88 (84-91)/86 (82-91)	28.17 ± 7.63/29.67 ± 7.73		800 IU daily	2 mo	2,7
Kerley, 2016 [32]	Ireland	RCT	17/22	10 (6-12)/7 (7-10)	19.6 (17-22)/18.2 (16-20)		19 (17-21)/17 (14.3-19)	96 (88-99)/94 (89-97)	22.17 ± 9.71/20.57 ± 7.93	39.86/20.63	2000 IU daily	15 weeks	3,4,7
Jensen, 2016 [33]	Canada	RCT	11/11	2.2 (1.9-3.5)/3.1 (2.1-3.9)					26.04 ± 4/24.04 ± 4.41	40.06 ± 5.21/32.85 ± 4	100 000 IU followed by 400 IU VD_3_ daily for 6 mo	6 mo	2
Martineau, 2015 [34]	UK	RCT	125/125	49.4 ± 14.8/46.4 ± 13.8		8/9	19.2 ± 3.9/18.9 ± 3.9		19.95 ± 10.1/19.79 ± 9.7	27.8 ± 8.41/18.63 ± 9.86	2-moly doses of 120 000 IU	12 mo	2,3
de Groot, 2015 [35]	Netherlands	RCT	22/22	59.0 ± 9.7/53.6 ± 16.7	26.6 ± 4.2/26.9 ± 4.8			92.5 ± 11.4/89.4 ± 12.8	24.71 ± 9.84/22.3 ± 9.52	91/48	single dose of 400 000 IU	9 weeks	3,4,12
Bar Yoseph, 2014 [36]	Israel	RCT	19/19	13.5 ± 3.6/12.4 ± 3.6	19.38 ± 3.29/21.53 ± 3.79	7/12			20.8 ± 6.5/20.0 ± 7.1	33.1 ± 7.9/20.0 ± 7.1	14 000 IU weekly	6 weeks	10,11,12
Castro, 2014 [38]	USA	RCT	201/207	39.9 ± 13.1/39.5 ± 12.7	32.00 ± 8.19/31.53 ± 9.51		19.0 (17.0-22.0)/20.0 (17.0-22.0)		19.8 ± 7.84/18.63 ± 7.69		100 000 IU once, then 4000 IU/d for 28 weeks	28 weeks	2
Arshi, 2014 [39]	Iran	RCT	64/66	24.40 (10.5-49.0)/28.64 (10.0-49.1) mean (range)	23.04 (16.5-35.5)/24.09 (15.64-38.0) mean (range)			75.8 ± 2.25/75.91 ± 3	23.82 ± 16.33/24.02 ± 16.45	91.57/23.43	100 000 IU, followed by 50 000 IU weekly	24 weeks	2,3,4
Yadav, 2014 [37]	India	RCT	50/50	9.15 ± 2.444/10.00 ± 1.876							60 000 IU per month for 6 mo	6 mo	2
Majak, 2011 [40]	Poland	RCT	24/24	10.8 ± 3.2/11.1 ± 3.3	18.5 ± 4.7/18.8 ± 3.5				36.1 ± 13.9/35.1 ± 16.9	37.6 ± 13.1/31.9 ± 12.1	500 IU daily	6 mo	2,3
Majak, 2009 [41]	Poland	RCT	18/18	<12					32.0 ± 3.1/31.3 ± 3.4	32.7 ± 2.5/30.3 ± 2.9	1000 IU daily	12 mo	3

SE – smoke exposure, BMI – body mass index, VD – vitamin D, RCT – randomized controlled trial, ACT/CACT – asthma control test/child asthma control test, EOT – end of treatment, m ± SD – mean ± standard deviation, mo – month

*1 – exacerbations for COPD, 2 – exacerbations for asthma, 3 – FEV1, 4 – FEV1/FVC, 5 – mMRC scores, 6 – SGRQ scores, 7 – ACT/CACT scores, 8 – length of hospital stay, 9 – mortality, 10 – cytokines, 11 – IgE, 12 – eosinophils.

### Exacerbation

#### Number of patients with exacerbation for COPD and asthma

**Figure 2:**
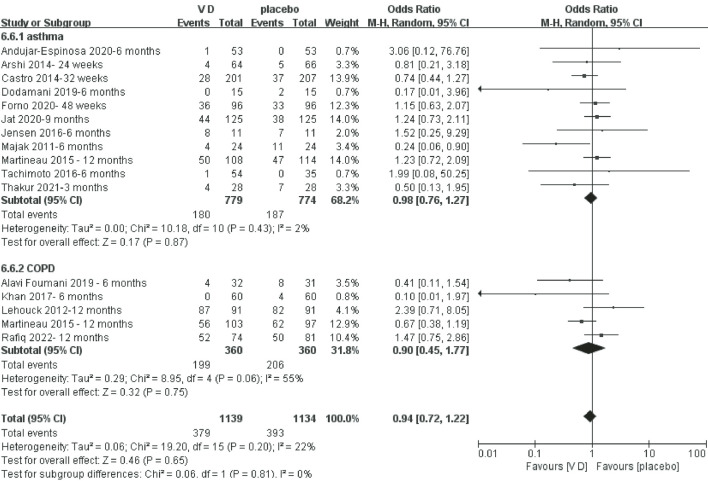
Meta-analysis of vitamin D (VD) supplementation on number of patients with exacerbation of chronic obstructive pulmonary disease (COPD) and asthma.

**Figure 2** shows that the number of patients with exacerbation of COPD and asthma in the VD supplementation group was not different from the comparator group.

#### Number of exacerbations of asthma

Figure S2 in the **Online Supplementary Document** shows that the number of exacerbations of asthma in the VD supplementation group was less compared with the comparator group, while there was no statistical difference (OR = 0.73, *P* = 0.06, *I^2^* = 59%).

### Pulmonary function

#### FEV1% change from baseline to end

**Figure 3:**
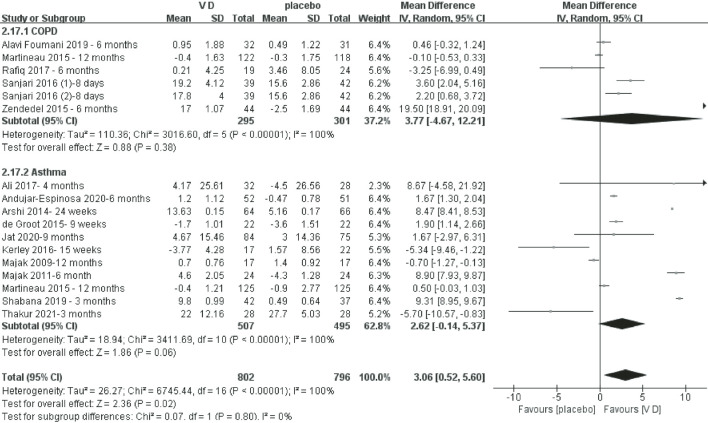
Meta-analysis of vitamin D (VD) supplementation on FEV1% change from baseline to end.

**Figure 3** shows that the VD supplementation group had a better recovery of FEV1% (OR = 3.06, *P* = 0.02, *I^2^* = 100%). In the COPD and asthma subgroup analysis, there was no significant difference.

#### FEV1/FVC change from baseline to end

Figure S3 in the **Online Supplementary Document** shows that there was no significant difference in FEV1/FVC changes between the VD supplementation group and control group from baseline to end (OR = 3.02, *P* = 0.06, *I^2^* = 99%). In the COPD subgroup analysis, there was no significant difference either. In the asthma subgroup analysis, the FEV1/FVC was significantly improved in the VD supplementation group (OR = 4.33, *P* = 0.02, *I^2^* = 99%).

### Questionnaire and rating scale

Figure S4 in the **Online Supplementary Document** shows that there was no significant difference in mMRC score changes between the VD supplementation group and control group from baseline to end. Figure S5 in the **Online Supplementary Document** shows that the SGRQ score was significantly improved in the VD supplementation group (OR = 2.97, *P* = 0.02, *I^2^* = 72%). Figure S6 in the **Online Supplementary Document** shows that the ACT score was not improved in the VD supplementation group.

### Length of hospital stay

Figure S7 in the **Online Supplementary Document** shows that the length of hospital stay was not changed in the VD supplementation group.

### Mortality

**Figure 4:**
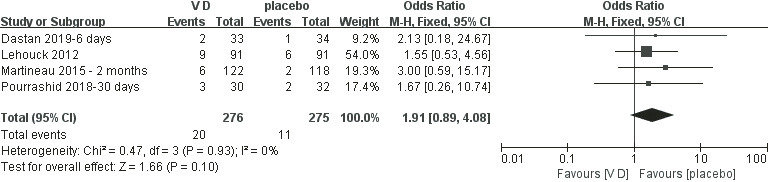
Meta-analysis of vitamin D (VD) supplementation on mortality of chronic obstructive pulmonary disease (COPD).

**Figure 4** shows that the mortality was not improved in the VD supplementation group.

### Inflammatory markers

Figure S8 and Figure S9 in the **Online Supplementary Document** show that the levels of IL-5 and Ig E were decreased in the VD supplementation group (OR = -9.18, *P* = 0.0004, *I^2^* = 99%; OR = -100.85, *P* < 0.00001, *I^2^* = 0%). However, Figures S10-S12 in the **Online Supplementary Document** reveal that the levels of IL-6 and IL-10, as well as eosinophil counts, were not significantly different between the VD supplementation group and placebo group.

In subgroup analysis based on serum VD, the IL-10 level of the VD deficiency group was significantly increased after the VD supplementation (OR = 2.51, *P* < 0.00001, *I^2^* = 32%). In VD sufficiency subgroup, there was no significant change after the VD supplementation. Subgroup analysis of IL-5, IL-6 and IL-10 based on types of diseases didn’t show significant difference.

## DISCUSSION

The present meta-analysis showed that VD supplementation had an effect on the control of certain indicators related to COPD and asthma. VD supplementation might affect pulmonary function, especially the FEV1% indicator. FEV1/FVC only improved in asthma. Quality of life and symptoms were improved only in COPD patient with improvement of SGRQ scores. VD supplementation might improve immune function since IL-5 and Ig E were decreased and IL-10 was increased in VD deficiency group after VD supplementary.

VD deficiency has long been associated with upper respiratory tract infection, and the exacerbation of COPD and asthma is also associated with infection [43]. Moreover, a cohort study has shown that smokers’ symptoms, lung function, and airway wall thickness improve after the VD supplementation [44]. It is known that cigarette smoking has a great effect on lung function and is also a risk factor for COPD. Therefore, it is reasonable to believe that VD deficiency is associated with the exacerbation of COPD and asthma. Many studies have reported that low VD levels are associated with the exacerbations of COPD and asthma. Therefore, many studies have aimed to control asthma and COPD by VD supplementation. However, the outcomes are quite different, and no convincing advice has been formed.

In our study with the latest reports, VD supplementation reduced the number of patients with exacerbations of COPD and asthma, while it was not statistically significant. Moreover, the total number of exacerbations decreased in the VD group (*P* = 0.06).

To avoid the influence of different baseline values, we calculated the difference between the final value and the initial value to compare the effect on pulmonary function parameters and the questionnaire rating scale. We found that VD significantly improved FEV1%. FEV1/FVC also tended to improve especially in asthmatic patients. The questionnaire rating scale also showed that VD supplementation improved the quality of life. At present, only SGRQ scores had a significant difference.

Cytokines are important markers of infection and immune status. IL-5 activation can lead to degranulation of eosinophils and cytotoxin release (such as IL-6), which can cause damage to surrounding cells and tissues [45]. Targeting IL-5 and IL-6 pathways are research hotspots in the treatment of asthma [46,47]. As an important anti-inflammatory cytokine, IL-10 is a promising candidate to control asthma [48]. In our present study, level of IL-5 significantly decreased in the VD supplementation group. However, baseline of IL-5 was very high in a trial consisting of 86 patients, leading to the significant decline of IL-5. The level of IL-6 decreased after the treatment, while there was no statistical significance. There was a significant increase in IL-10 in VD-deficient patients after the treatment, while the effect was not obvious in patients with VD sufficiency. Besides IL-10, we also analysed other indicators and found that VD supplementation did not affect the indicators no matter the VD baseline level was higher or lower than 20 ng/mL.

It has been shown that a high serum level of total IgE and eosinophil counts are predisposing factors of allergic asthma [49,50]. A study consisting of 100 children has shown that the VD level is negatively correlated with serum IgE levels [51]. Besides, asthmatic children with serum level of 25(OH)D<24 ng/ml have higher eosinophil counts and IgE levels [52]. In our present work, we found that there was a significant decrease in IgE, while no significant change in eosinophil counts was observed.

There are many studies on relationship between VD and asthma or COPD, while the results are quite different. These differences may be attributed to the reasons as follows. First, genetic variants in the VD pathway affect serum levels of VD, thus affecting atopy and asthma [53]. Second, an experiment has shown that after the VD supplementation, the level of serum 25(OH)D in patients with asthma and COPD increase slowly. Gene expression analysis shows that the metabolic capacity of VD decreased under such diseased condition [54]. Another research shows that even under seasonal oral VD supplementation, patients with a positive history of an asthma attack in the previous 4 weeks present significantly lower serum 25(OH)D concentrations compared with their peers with no disease exacerbation [55]. Therefore, VD deficiency in asthma and COPD may be a chicken or egg story [56]. Third, studies have shown that plasma VD is also related to the content of unsaturated fatty acids in blood, which is a possible regulatory pathway. It may also be the reason for poor outcomes for single use of VD to control inflammation in some people [57]. Taken together, it is not very clear how VD affects respiratory system. Genetic analysis has found that maternal 17q21 genotype has an important influence on the protective effects of prenatal VD supplementation against offspring asthma/recurrent wheeze [58]. Besides, the acute wheeze-specific gene module shows a correlation with VD and asthma medication [59]. Some studies have investigated the effect of VD supplementation on the mother with asthmatic history during pregnancy. It seems that sufficient serum 25(OH)D can reduce the risk of asthma in offspring born to asthmatic mothers [60]. Except that, COPD reveals no impact of VD on known molecular pathways.

Considering the risk of fracture and metabolism, the International Osteoporosis Foundation recommends 600 IU VD per day in younger adults and 800 IU per day in older adults to reach a status with 25(OH)D levels of 20 ng/mL [61]. Based on current research, patients with asthma and COPD might be accompanied by low VD status [8]. VD supplementation should be supplemented even if it had no significant effect on disease control.

Our study has several limitations. First, some trials included in this review differed in their definition of exacerbations. Some defined exacerbations as sustained worsening of symptoms and requiring drug intervention, while others were defined according to the pulmonary function, such as FEV1. Second, the lack of original data in the studies limited our analysis. We had to calculate some continuous variables based on the Cochran handbook and published methods. Third, because of large differences in usage in clinical trials presented in the included RCTs, the optimal dosage and duration of VD supplementation are yet unknown.

## CONCLUSIONS

VD supplementation improved the indicators of asthma and COPD, especially in pulmonary function, SGRQ scores, IL-5, IgE, and IL-10 (in serum VD deficiency group). Although the treatment effect was heterogeneous across trials and might have been overestimated, VD supplementation was a low-cost, low-risk, promising method to control asthma and COPD. More investigations are required to guide the dosage to achieve a better effect.

## Additional material


Online Supplementary Document
