# Supplementary material for: Efficacy of vitamin D supplementation on COPD and asthma control: A systematic review and meta-analysis
Source: J Glob Health. 2022 Dec 16;12:04100. doi: 10.7189/jogh.12.04100 (PMC9754066; doi:10.7189/jogh.12.04100)
Supplement: Online Supplementary Document [file jogh-12-04100-s001.pdf]

ONLINE SUPPLEMENTARY DOCUMENT

Title: Efficacy of vitamin D supplementation on COPD and asthma control: a systematic review and meta-analysis

Authors:Yuhang Wang, Jin Wang, Li Chen, Huan Zhang, Ling Yu, Yulong Chi, Mengli Chen, Yun Cai

Fig. S1 Risk of bias assessed by the Cochrane assessment tool

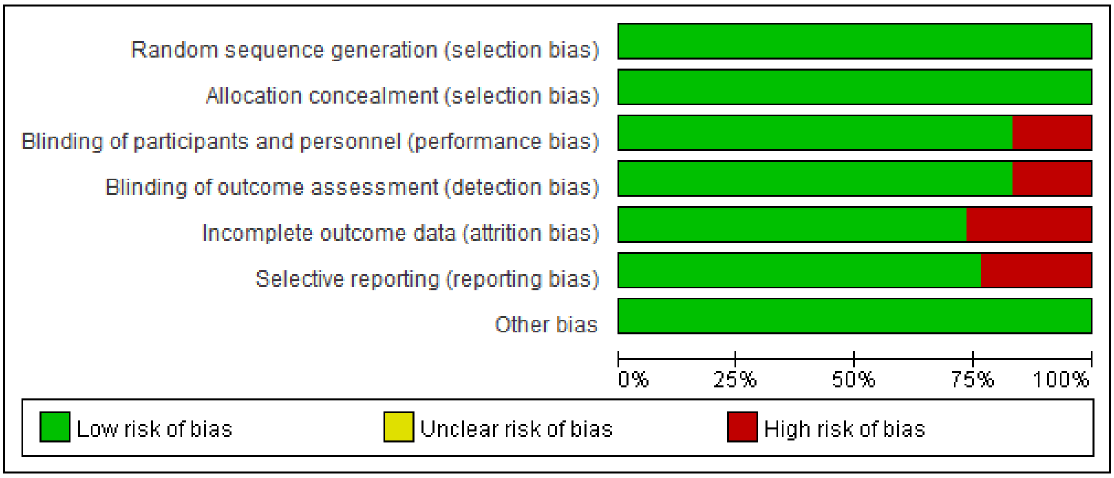

Fig. S2 Meta-analysis of VD supplementation on number of exacerbations of asthma

	V D		placebo			Odds Ratio
Study or Subgroup	Events	Total	Events	Total	Weight	M-H, Random, 95% CI
Andujar-Espinosa 2020-6 months	12	318	33	318	13.4%	0.34 [0.17, 0.67]
Castro 2014-32 weeks	28	1608	37	1656	18.0%	0.78 [0.47, 1.27]
Dodamani 2019-6 months	0	90	2	90	1.2%	0.20 [0.01, 4.13]
Forno 2020- 48 weeks	56	1152	49	1152	21.0%	1.15 [0.78, 1.70]
Jensen 2016-6 months	7	66	8	66	7.4%	0.86 [0.29, 2.53]
Martineau 2015-12 months asthma	136	1500	145	1500	25.4%	0.93 [0.73, 1.19]
Yadav 2014-6 months	14	200	29	200	13.6%	0.44 [0.23, 0.87]
**Total (95% CI)**		**4934**		**4982**	**100.0%**	**0.73 [0.52, 1.02]**
Total events	253		303			

Heterogeneity: Tau^2 = 0.10; Chi^2 = 14.47, df = 6 (P = 0.02); I^2 = 59%
Test for overall effect: Z = 1.86 (P = 0.06)

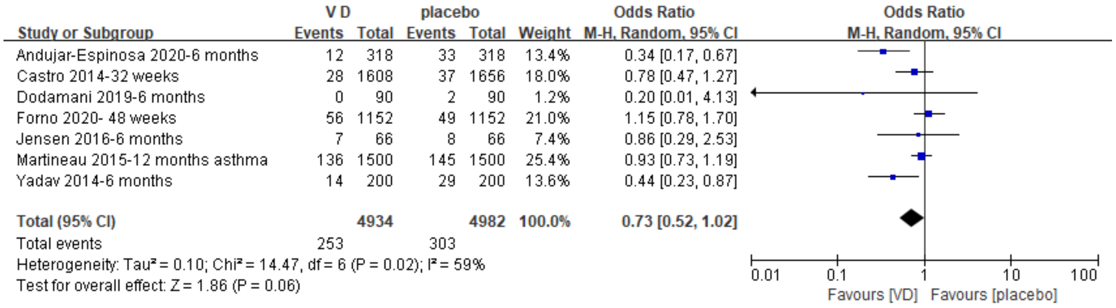

The total number is the number of patients multiplied by the month of observation. Abbreviations: VD, vitamin D.

Fig. S3 Meta-analysis of VD supplementation on FEV1/FVC change from baseline to end

	V D			placebo				Mean Difference
Study or Subgroup	Mean	SD	Total	Mean	SD	Total	Weight	IV, Random, 95% CI
**2.18.1 COPD**								
Alavi Foumani 2019 - 6 months	0.99	1.28	32	0.45	1.51	31	9.9%	0.54 [-0.15, 1.23]
Rafiq 2017 - 6 months	-0.34	3.02	24	0.99	4.38	26	9.6%	-1.33 [-3.40, 0.74]
Sanjari 2016 (1)-8 days	4.9	3.3	39	2.7	3.37	42	9.8%	2.20 [0.75, 3.65]
Sanjari 2016 (2)-8 days	5.3	2.68	39	2.7	3.37	42	9.8%	2.60 [1.28, 3.92]
**Subtotal (95% CI)**			**134**			**141**	**39.1%**	**1.11 [-0.32, 2.54]**
Heterogeneity: Tau^2 = 1.62; Chi^2 = 14.80, df = 3 (P = 0.002); I^2 = 80%								
Test for overall effect: Z = 1.52 (P = 0.13)								
**2.18.2 asthma**								
Ali 2017- 4 months	-0.33	28.71	32	-5.17	35.93	28	2.6%	4.84 [-11.78, 21.46]
Andujar-Espinosa 2020-6 months	10.07	0.96	52	-0.78	1.08	51	10.0%	10.85 [10.46, 11.24]
Arshi 2014- 24 weeks	6.35	2.91	64	3.32	3.76	66	9.8%	3.03 [1.88, 4.18]
de Groot 2015- 9 weeks	0	1.4	22	-3.4	2.1	22	9.9%	3.40 [2.35, 4.45]
Jat 2020-9 months	-1.67	9.05	84	-2.5	6.8	75	9.4%	0.83 [-1.64, 3.30]
Kerley 2016- 15 weeks	1	5.09	17	0	3.17	22	9.3%	1.00 [-1.76, 3.76]
Shabana 2019 - 3 months	4.8	2.1	42	-1.35	1.28	37	9.9%	6.15 [5.39, 6.91]
**Subtotal (95% CI)**			**313**			**301**	**60.9%**	**4.33 [0.84, 7.82]**
Heterogeneity: Tau^2 = 19.02; Chi^2 = 414.76, df = 6 (P < 0.00001); I^2 = 99%								
Test for overall effect: Z = 2.43 (P = 0.02)								
**Total (95% CI)**			**447**			**442**	**100.0%**	**3.02 [-0.13, 6.17]**

Heterogeneity: Tau^2 = 25.88; Chi^2 = 976.27, df = 10 (P < 0.00001); I^2 = 99%
Test for overall effect: Z = 1.88 (P = 0.06)
Test for subgroup differences: Chi^2 = 2.79, df = 1 (P = 0.09), I^2 = 64.2%

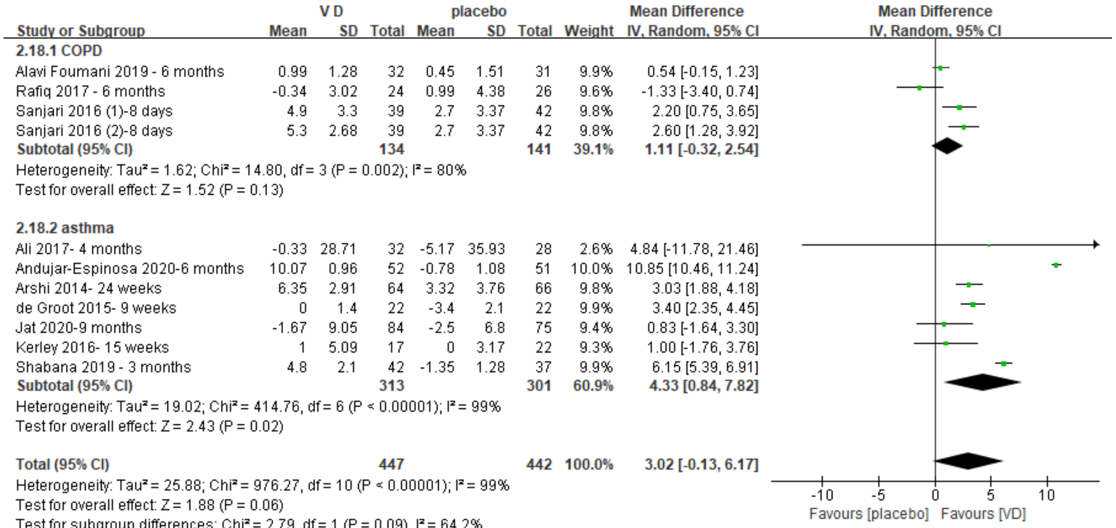

Abbreviations: VD, vitamin D; COPD, chronic obstructive pulmonary disease.

Fig. S4 Meta-analysis of VD supplementation on mMRC score change from baseline to end of COPD

	V D			placebo				Mean Difference
Study or Subgroup	Mean	SD	Total	Mean	SD	Total	Weight	IV, Fixed, 95% CI
Dastan 2019-6 days	-0.97	0.86	33	-0.82	0.81	34	47.9%	-0.15 [-0.55, 0.25]
Pourrashid 2018-120 days	-1.27	0.78	30	-1	0.76	32	52.1%	-0.27 [-0.65, 0.11]
**Total (95% CI)**			**63**			**66**	**100.0%**	**-0.21 [-0.49, 0.06]**

Heterogeneity: Chi² = 0.18, df = 1 (P = 0.67); I² = 0%
Test for overall effect: Z = 1.50 (P = 0.13)

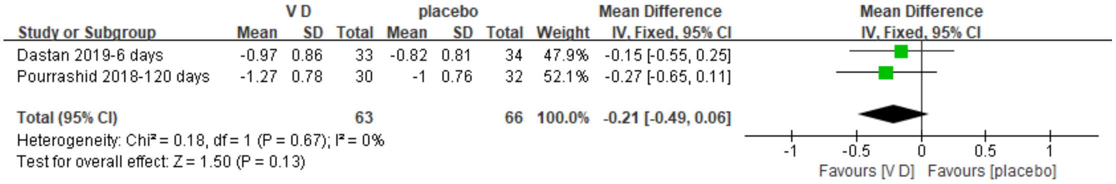

Abbreviations: Mmrc, Modified Medical Research Council; VD, vitamin D; COPD, chronic obstructive pulmonary disease.

Fig. S5 Meta-analysis of VD supplementation on SGRQ score change from baseline to end of COPD

	V D			placebo				Mean Difference
Study or Subgroup	Mean	SD	Total	Mean	SD	Total	Weight	IV, Random, 95% CI
Bjerk 2013-6 weeks	1.3	5.6	18	-1	6.9	18	20.8%	2.30 [-1.81, 6.41]
Martineau 2015 - 12 months	-1	7.67	122	-2.4	8.36	118	36.9%	1.40 [-0.63, 3.43]
Pourrashid 2018-120 days	8.66	3.32	30	3.99	2.19	32	42.4%	4.67 [3.26, 6.08]
**Total (95% CI)**			**170**			**168**	**100.0%**	**2.97 [0.51, 5.43]**

Heterogeneity: Tau² = 3.20; Chi² = 7.08, df = 2 (P = 0.03); I² = 72%
Test for overall effect: Z = 2.37 (P = 0.02)

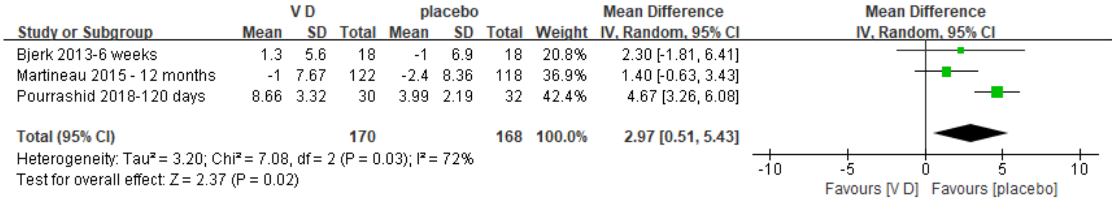

Abbreviations: SGRQ, St George's Respiratory Questionnaire; VD, vitamin D; COPD, chronic obstructive pulmonary disease.

Fig. S6 Meta-analysis of VD supplementation on ACT score change from baseline to end of asthma

	V D			placebo				Mean Difference
Study or Subgroup	Mean	SD	Total	Mean	SD	Total	Weight	IV, Random, 95% CI
Jat 2020-9 months	2.33	3.76	112	1.83	3.01	108	45.7%	0.50 [-0.40, 1.40]
Kerley 2016- 15 weeks	1.33	4.85	17	2.83	3.96	22	12.1%	-1.50 [-4.34, 1.34]
Tachimoto 2016-6 months	1.4	2.44	52	0	2.27	34	42.2%	1.40 [0.39, 2.41]
**Total (95% CI)**			**181**			**164**	**100.0%**	**0.64 [-0.46, 1.73]**

Heterogeneity: Tau² = 0.47; Chi² = 4.30, df = 2 (P = 0.12); I² = 53%
Test for overall effect: Z = 1.14 (P = 0.25)

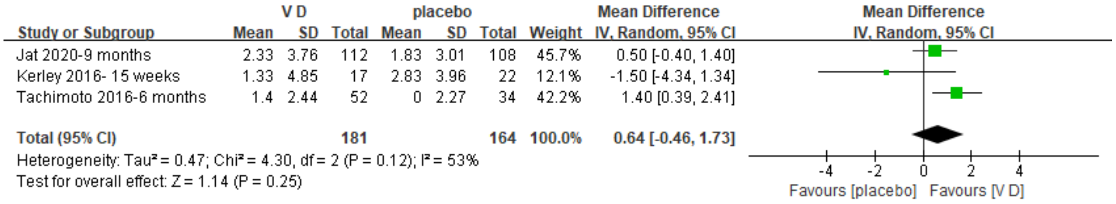

Abbreviations: ACT, asthma control test; VD, vitamin D.

Fig. S7 Meta-analysis of VD supplementation on length of hospital stay of COPD

	V D			placebo				Mean Difference
Study or Subgroup	Mean	SD	Total	Mean	SD	Total	Weight	IV, Fixed, 95% CI
Dastan 2019-6 days	7.48	3.02	33	8.09	3.38	34	49.4%	-0.61 [-2.14, 0.92]
Pourrashid 2018-30 days	7.37	2.59	30	8.06	3.46	32	50.6%	-0.69 [-2.21, 0.83]
**Total (95% CI)**			**63**			**66**	**100.0%**	**-0.65 [-1.73, 0.43]**

Heterogeneity: Chi² = 0.01, df = 1 (P = 0.94); I² = 0%
Test for overall effect: Z = 1.18 (P = 0.24)

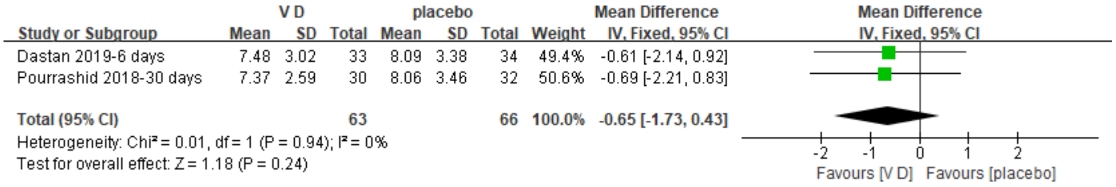

Abbreviations: VD, vitamin D; COPD, chronic obstructive pulmonary disease.

Fig. S8 Meta-analysis of VD supplementation on IL-5

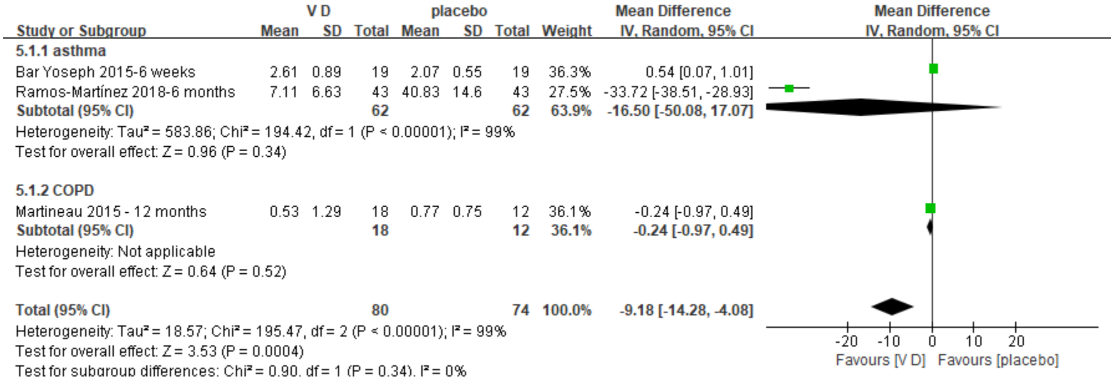

Abbreviations: VD, vitamin D; COPD, chronic obstructive pulmonary disease.

Fig. S9 Meta-analysis of VD supplementation on IgE of asthma

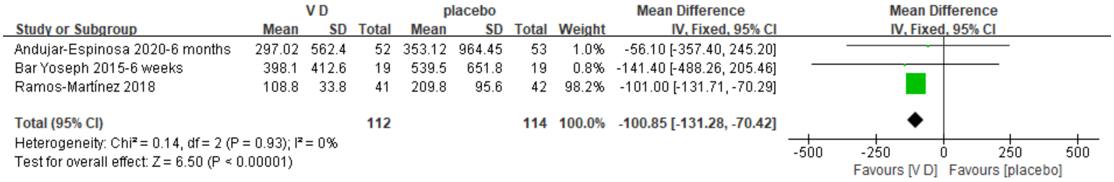

Abbreviations: VD, vitamin D.

Fig. S10 Meta-analysis of VD supplementation on IL-6

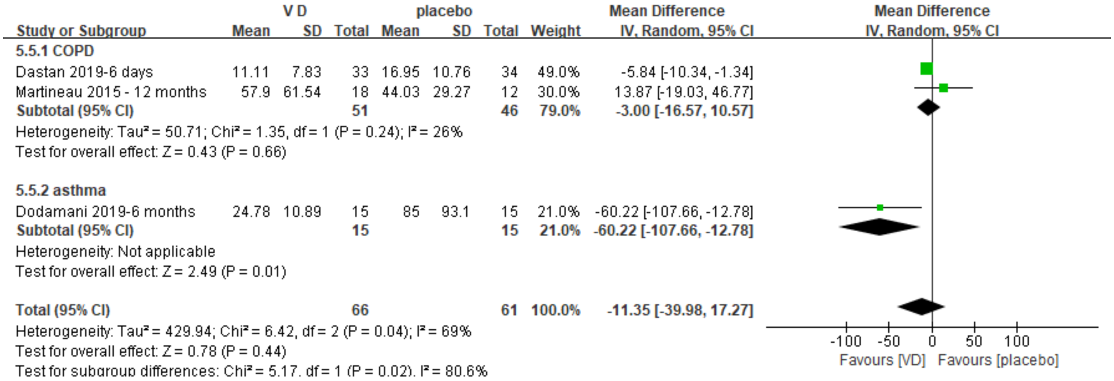

Abbreviations: VD, vitamin D; COPD, chronic obstructive pulmonary disease.

Fig. S11 Meta-analysis of VD supplementation on IL-10

Study or Subgroup	VD Mean	VD SD	VD Total	placebo Mean	placebo SD	placebo Total	Weight	Mean Difference IV, Random, 95% CI
**5.7.1 ashtma**								
Bar Yoseph 2015-6 weeks	1.27	0.45	19	1.53	0.53	19	20.2%	-0.26 [-0.57, 0.05]
Dodamani 2019-6 months	12.98	8.46	15	13.7	9.03	15	19.5%	-0.72 [-6.98, 5.54]
Ramos-Martínez 2018-6 months	25.52	0.43	43	0.6	0.83	43	20.2%	24.92 [24.64, 25.20]
Shabana 2019 - 3 months	9.06	2.44	42	6.28	2.07	37	20.1%	2.78 [1.79, 3.77]
**Subtotal (95% CI)**			**119**			**114**	**79.9%**	**6.74 [-10.00, 23.49]**
Heterogeneity: Tau² = 289.54; Chi² = 14320.56, df = 3 (P < 0.00001); I² = 100%								
Test for overall effect: Z = 0.79 (P = 0.43)								
**5.7.2 COPD**								
Martineau 2015 - 12 months	1.97	4.02	18	0.6	1.51	12	20.1%	1.37 [-0.67, 3.41]
**Subtotal (95% CI)**			**18**			**12**	**20.1%**	**1.37 [-0.67, 3.41]**
Heterogeneity: Not applicable								
Test for overall effect: Z = 1.31 (P = 0.19)								
**Total (95% CI)**			**137**			**126**	**100.0%**	**5.67 [-9.18, 20.51]**
Heterogeneity: Tau² = 284.45; Chi² = 14449.32, df = 4 (P < 0.00001); I² = 100%								
Test for overall effect: Z = 0.75 (P = 0.45)								
Test for subgroup differences: Chi² = 0.39, df = 1 (P = 0.53), I² = 0%								

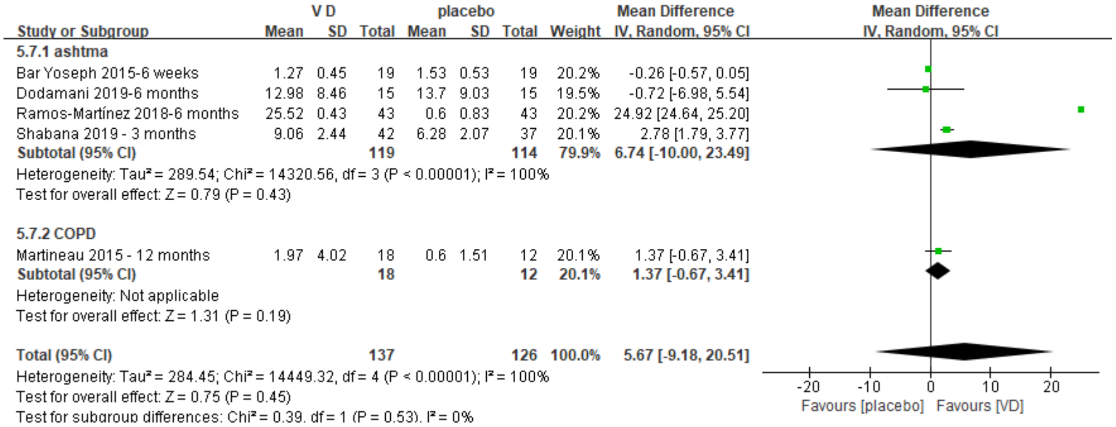

Study or Subgroup	VD Mean	VD SD	VD Total	placebo Mean	placebo SD	placebo Total	Weight	Mean Difference IV, Fixed, 95% CI
**5.6.1 VD baseline >20ng/ml**								
Bar Yoseph 2015-6 weeks	1.27	0.45	19	1.53	0.53	19	88.9%	-0.26 [-0.57, 0.05]
Dodamani 2019-6 months	12.98	8.46	15	13.7	9.03	15	0.2%	-0.72 [-6.98, 5.54]
**Subtotal (95% CI)**			**34**			**34**	**89.1%**	**-0.26 [-0.57, 0.05]**
Heterogeneity: Chi² = 0.02, df = 1 (P = 0.89); I² = 0%								
Test for overall effect: Z = 1.64 (P = 0.10)								
**5.6.2 VD baseline <20ng/ml**								
Martineau 2015 - 12 months	1.97	4.02	18	0.6	1.51	12	2.1%	1.37 [-0.67, 3.41]
Shabana 2019 - 3 months	9.06	2.44	42	6.28	2.07	37	8.8%	2.78 [1.79, 3.77]
**Subtotal (95% CI)**			**60**			**49**	**10.9%**	**2.51 [1.62, 3.40]**
Heterogeneity: Chi² = 1.48, df = 1 (P = 0.22); I² = 32%								
Test for overall effect: Z = 5.50 (P < 0.00001)								
**Total (95% CI)**			**94**			**83**	**100.0%**	**0.04 [-0.25, 0.33]**
Heterogeneity: Chi² = 34.37, df = 3 (P < 0.00001); I² = 91%								
Test for overall effect: Z = 0.27 (P = 0.79)								
Test for subgroup differences: Chi² = 32.87, df = 1 (P < 0.00001), I² = 97.0%								

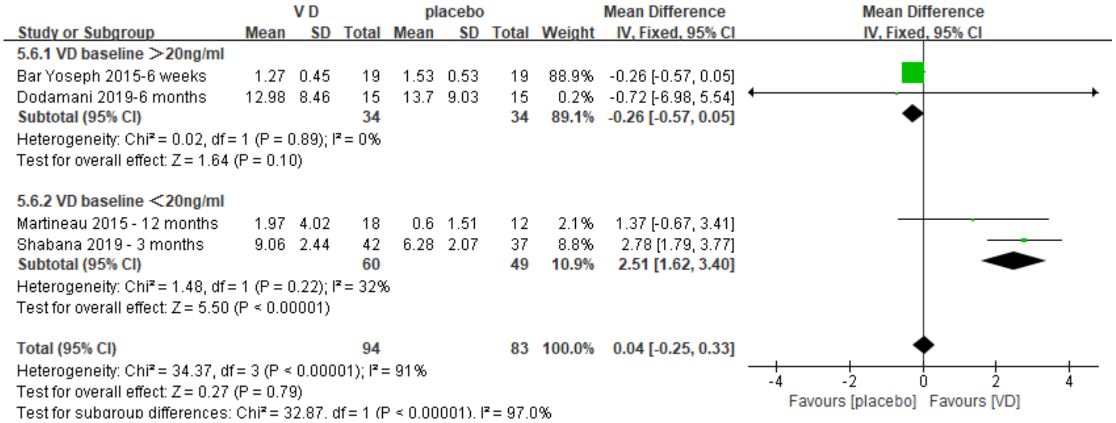

The research of Ramos-Martinez didn't include the character of VD baseline.
Abbreviations: VD, vitamin D; COPD, chronic obstructive pulmonary disease.

Fig. S12 Meta-analysis of VD supplementation on eosinophils of asthma

Study or Subgroup	VD Mean	VD SD	VD Total	placebo Mean	placebo SD	placebo Total	Weight	Mean Difference IV, Fixed, 95% CI
Bar Yoseph 2015-6 weeks	0.32	0.18	19	0.31	0.25	19	42.6%	0.01 [-0.13, 0.15]
de Groot 2015- 9 weeks	0.2	0.16	22	0.23	0.24	22	56.3%	-0.03 [-0.15, 0.09]
Ramos-Martínez 2018	0.77	1.3	43	1.29	2.5	43	1.2%	-0.52 [-1.36, 0.32]
**Total (95% CI)**			**84**			**84**	**100.0%**	**-0.02 [-0.11, 0.07]**
Heterogeneity: Chi² = 1.56, df = 2 (P = 0.46); I² = 0%								
Test for overall effect: Z = 0.40 (P = 0.69)								

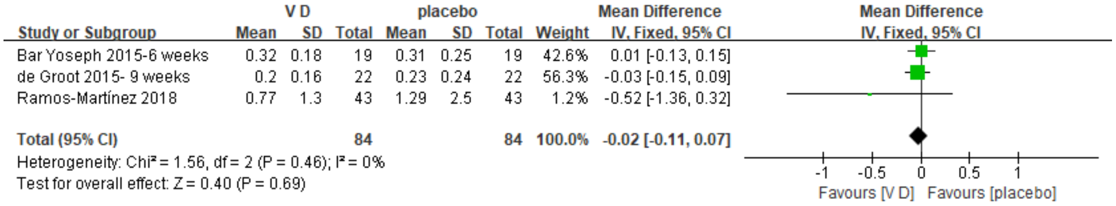

Abbreviations: VD, vitamin D.
